# A parasitic or mutualistic conundrum: can symbiotic protists increase thermal tolerance in a semi-aquatic insect?

**DOI:** 10.1098/rsos.251061

**Published:** 2025-09-03

**Authors:** Md Tangigul Haque, Shatabdi Paul, Marie E. Herberstein, Md Kawsar Khan

**Affiliations:** ^1^ School of Natural Sciences, Macquarie University, Sydney, New South Wales, Australia; ^2^ Centre for Taxonomy and Morphology, Leibniz Institute for the Analysis of Biodiversity Change, Hamburg, Germany; ^3^ Department of Biology, University of Hamburg, Hamburg, Germany; ^4^ Department of Pharmacy, Chemistry and Biology, Free University of Berlin, Berlin, Germany; ^5^ Applied BioScience, Macquarie University, Sydney, New South Wales, Australia

**Keywords:** global climate change, gregarine endosymbionts, endoparasites, thermal tolerance, insect conservation

## Abstract

Rising temperatures and frequent heatwaves pose a major threat to ectotherms due to their reliance on environmental temperature for physiological processes. Thermal tolerance, the ability to withstand varying temperature, determines how effectively and efficiently individuals can survive under extreme conditions. Host–microbial symbiotic interactions can influence thermal tolerance in insects; however, we have limited information especially for some endosymbionts such as gregarines, a group of apicomplexan endoparasites, which are commonly found in the guts of many aquatic and terrestrial insects. Gregarines are often considered parasitic, while a few recent studies have shown beneficial effects on hosts. Here, we tested the impact of gregarines on thermal tolerance in *Ischnura heterosticta* damselflies. We found that damselflies naturally infected with gregarines had higher thermal tolerance than damselflies without gregarine infections. Our findings provide evidence in support of gregarines as an endosymbiont of *I. heterosticta* damselfly. Our study indicates that gregarine endosymbionts may assist damselfly and possibly other semi-aquatic insects to sustain extreme heat and highlights the importance of understanding host–symbiont interactions in the context of climate change and species conservation.

## 1. Introduction

Global change, especially anthropogenic climate change, is contributing to the decline of insects worldwide [1,2]. Semi-aquatic insects, such as damselfly and dragonfly, are experiencing a significant rate of biodiversity loss, with projections estimating a ~33% decline over the coming decades [2]. This decline is largely attributed to thermal stress causing direct and indirect mortality [3], facilitated by the rising average global temperature, and more frequent and intense heat waves [4]. Understanding the mechanisms by which insects cope with thermal stress is crucial, yet limited, in order to determine species and populations vulnerability to heat events.

Insects deal with thermal stress by physiological and behavioural responses [5,6] as well as through interactions with symbiotic organisms [7]. Many insects harbour bacterial, fungal and protists symbionts, which have positive impacts on reproduction, metabolism and may also influence insect thermal tolerance [8]. The potential of endosymbionts impacting thermal tolerance is complex and can vary depending on host–endosymbiont pairing. For instance, endosymbiotic bacteria in the oriental fruit fly, *Bactrocera dorsalis*, are associated with reduced thermal stress [9]. Conversely, variation in the bacterial symbiont *Buchnera aphidicola* may limit thermal tolerance in black bean aphid (*Aphis fabae*)*,* blue alfalfa aphid (*Acyrthosiphon kondoi*) and pea aphid (*Acyrthosiphon pisum*) [8]. This complexity arises from potential trade-offs between the host’s ability to tolerate thermal stress and its susceptibility to infection, leading to unpredictable outcomes at ecological and evolutionary scales [10]. Despite the importance of understanding thermal tolerance in the face of climate change, the role of endosymbionts in mitigating insect thermal tolerance remains largely underappreciated especially in semi-aquatic insects, albeit prime importance given the increased extinction risk these insects face under thermal stress [11,12].

Gregarines are ubiquitous endo-parasites found in the guts of marine, freshwater and terrestrial invertebrates [13]. Gregarines can be mutualists, commensals and parasites to hosts [14]. For example, gregarine infection reduced larval and pupal development of red flour beetle *Tribolium castaneum* [15], whereas gregarine-infected cat flea larvae (*Ctenocephalides felis*) developed and emerged faster than non-infected individuals [16]. Odonata (damselfly and dragonfly) are frequently infected with gregarines, which are often considered parasitic [17]. Gregarines may reduce damselflies survivability, muscle protein composition and mating [18–20]. For example, infection with gregarine endoparasites (genus *Hoplorhynchus*) reduced flight muscle performance in dragonfly *Libellula pulchella* [20]. Though we have not found evidence of a direct link between gregarine apicomplexans and insects thermal tolerance, there is evidence that other endosymbionts such as *Rickettsiella grylli* can increase host thermal preference [21]. Hence, it is likely that gregarine endoparasites may also enhance thermal function of insects during temperature stress.

In this study, we aim to determine the impact of gregarine infections on thermal tolerance in *Ischnura heterosticta* damselfly. We assessed thermal tolerance via time to heat stupor (the duration an individual can tolerate at a given temperature before knockdown due to heat stress) in naturally gregarine-infected and non-infected damselflies. We predicted that if gregarines are parasitic, damselflies naturally harbouring gregarines would have lower thermal tolerance compared with non-infected individuals; if gregarines are commensals, there would be no change in thermal tolerance between infected and non-infected individuals; and if gregarines are mutualists, infected individuals would have higher thermal tolerance than non-infected ones.

## 2. Methods

We captured damselflies from a pond located at Macquarie University (33.772° S, 151.114° E) in North Ryde, Australia in November 2024 using an insect sweep net applying a previously established method [11,22]. We captured a total 223 *I. heterosticta* damselflies (male = 119 and female = 104) on sunny and partially sunny days (average temperature during collection days was 26.8 ± 0.29°C). Within 20 min of collection, we transported captured damselflies to the laboratory using round mesh travel containers (14 cm diameter × 23 cm height).

We kept the damselflies at room temperature for 15–20 min before performing the experiment. The damselflies were introduced into the pre-heated (41°C) 15 ml centrifuge tubes (Sarstedt AG & Co. KG, Nümbrecht, Germany) head first, and the tubes were then placed vertically in a water bath with the lids positioned at the top. We submerged the centrifuge tubes in the water bath below the water level around three to four cm to ensure uniform heating. Inside the tubes, damselflies were settled naturally towards the lower part of the tubes. Although we pre-heated centrifuge tubes to 41°C, we waited approximately 2–3 min after transferring damselflies into the tubes before placing the tubes into the water bath to allow the internal temperature of the centrifuge tubes to stabilize at 41°C before starting the experiment. Then, we measured time to heat stupor at a constant temperature of 41°C in a water bath (model: MyBath mini water bath, Benchmark Scientific B2000-4-T5; accuracy: ± 0.5°C) and recorded the duration of time before knockdown.

During the experiment, we kept centrifuge tube lids open to allow air exchange, prevent oxygen limitation and facilitate observation. We determined the endpoint of the experiment by observing the loss of coordinated movement in the damselflies by gentle moving the tubes. After opening the water bath lid, we observed the condition of damselflies quickly to minimize heat loss. After assessing the time to heat stupor, damselflies were kept in −30°C until we dissected their gut to check for the presence of gregarine endoparasites using a compound microscope. We scored presence or absence of gregarines for each individual. As we determined the presence or absence of gregarines using visual inspection only, it is possible that some positive specimens went undetected if the infection rate was very low, potentially underestimating the actual endoparasite prevalence.

We applied generalized linear mixed models using template model builder (glmmTMB) to assess the impact of gregarine endosymbionts on infected and non-infected damselfly thermal tolerance [23]. We fitted our model using time to heat stupor as a response variable and sex as a fixed factor to observe the impact of gregarines on different sexes of damselfly thermal tolerance. In a separate model, we used time to heat stupor as the response variable and endoparasite status as a fixed factor to determine the impact of gregarines on the thermal tolerance of infected and non-infected individuals. We checked the residual plots and goodness-of-fits of our models using DHARMa package in R [24]. We used DurgaDiff function of ‘Durga’ R package [25] to determine to what extent time to heat stupor varies between gregarine-infected and non-infected damselflies. The DurgaDiff function estimated the confidence intervals of the mean by bootstrapping the data 1000 times. Both the DurgaDiff function and the glmmTMB model used in our study did not assume normality of the data. We performed all analyses in R version 4.1.2 [26]. All data were reported in mean ± s.e.

## 3. Results

**Figure 1.**
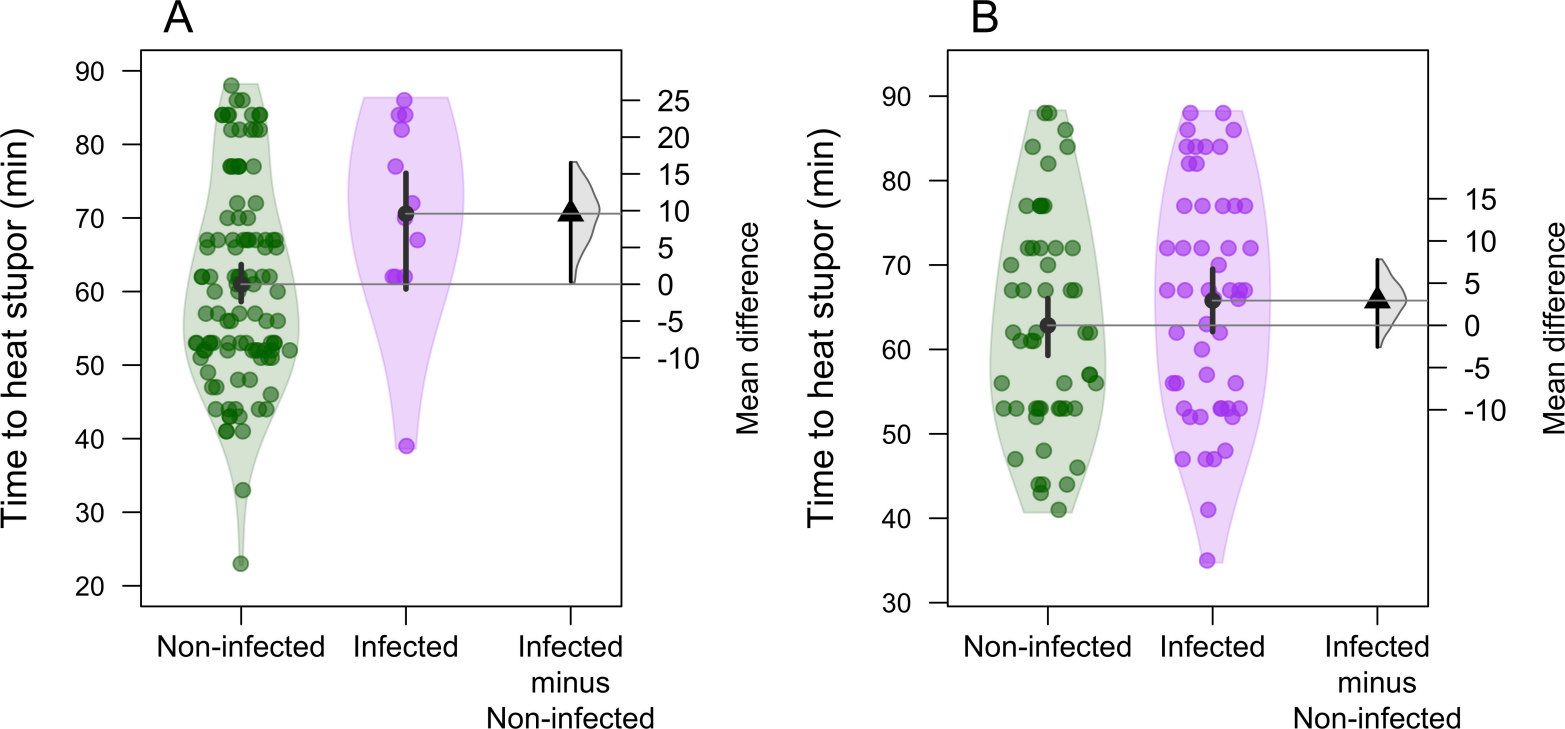
DurgaPlot showing gregarine-infected (A) males and (B) females damselflies had higher time to heat stupor than non-infected damselflies. The black circle denotes mean, vertical bars indicate 95% confidence interval of mean, and each green and purple dot represent time to heat stupor of an individual. On the right side of each graph, the triangle represents mean differences in time to heat stupor in infected and non-infected individuals, and the vertical line indicates the 95% CI of mean difference, and the half violin indicates distribution of mean difference.

Average time to heat stupor for *I. heterosticta* was 63 ± 0.9 min (pooling all individuals). Females (*n* = 104; mean = 64.28 ± 1.29 min) had longer time to heat stupor (glmmTMB: estimate = 2.33 ± 1.81, Z = 1.29, *p* = 0.19; mean diff = 2.33; 95% CI [−1.05, 5.96]) than males (*n* = 119; mean = 61.95 ± 1.26 min), although the result was not significant. Gregarine prevalence was higher in females (49.03%) than males (10.08%). Gregarine-infected males had a greater time to heat stupor (*n* = 12, mean = 70.5 ± 3.8 min) than gregarine-free males (*n* = 107, mean = 60.9 ± 1.3 min) (glmmTMB: estimate = 9.53 ± 4.1, Z = 2.34, *p* = 0.01; mean difference = 9.5, 95% CI [1.34, 16.94]; figure 1A). Similarly, gregarine-infected females had a greater but non-significant time to heat stupor (*n* = 51, mean = 65.78 ± 1.9 min) than non-infected females (*n* = 53, mean = 62.84 ± 1.7 min) (glmmTMB: estimate = 2.93 ± 2.56, Z = 1.14, *p* = 0.25; mean difference = 2.93, 95% CI [−2.82, 7.76]; figure 1B).

## 4. Discussion

Here, we demonstrated that gregarine infection did not reduce thermal tolerance in *I. heterosticta* damselfly; if anything, it was positively correlated with increased thermal tolerance in males, although experimental manipulation would be required to establish causality. In both males and females, we found that gregarine-infected individuals had higher thermal tolerance than non-infected individuals; however, the trend was non-significant in females. Our study provides evidence for the assumption that gregarines may function as a mutualistic endosymbiont for semi-aquatic insects, a pattern that previously found in other taxa including cat flea (*C. felis*), where gregarine infection accelerated larval development and earlier emergence [16]. Similarly in *Aedes triseriatus* mosquitoes, infection with the gregarine parasite *Ascogregarina barrette* increased anti-predatory behaviour in mosquito larvae, which reduced larval mortality when the predator *Toxohrynchites rutilus* was present [27].

While many studies previously described the parasitic role of gregarines in various insect species [20,28,29], our study provides evidence that gregarines may have a beneficial impact on the thermal tolerance in insects. Specifically, our findings in damselflies suggest that gregarines may enhance host thermal tolerance, similar to the role of bacterial endosymbionts (e.g. *Acinetobacter*, *Brevibacillus*, *Bacillus*, *Buchnera*, *Enterobacter*, *Enterococcus*, *Pseudomonas* and *Staphylococcus*) in facilitating thermal tolerance in other insects such as flies and aphids [9,30]. However, we do not know if gregarines provide other benefits for damselflies, such as immunity, nutrition and reproduction as have been shown previously for other insect–symbiont relationship. The possible mechanisms by which gregarines may delay time to heat stupor in hosts could be measured by enhancing host heat shock gene expression (e.g. hsp70 gene), which reduces heat-induced cell damage [31–33] or indirectly by affecting secondary symbionts [32], or modulation of host behaviour adjustments [34].

Since we did not experimentally infect damselflies, our data could also reflect that gregarines infect damselflies with naturally high thermal tolerance. While we cannot entirely dismiss this explanation, we can test the likelihood of survivor bias by looking at the variance in time to heat stupor between infected and uninfected individuals. If gregarines preferentially infect individuals with higher thermal tolerance, we expect a bimodal distribution of time to heat stupor in the uninfected individuals: low thermal tolerance individuals and the not-yet-infected high thermal tolerance individuals. However, when we compare the variance between infected and noninfected individuals, we see no difference (male: *F*-test, *F* = 0.98, df = 11, *p* = 0.92; female: *F* = 1.12, df = 50, *p* = 0.67). Nevertheless, in a different study [35], gregarine-infected damselflies were in better condition than uninfected individuals. These data could either support survivor bias or indicate that gregarines provide nutritional benefits to their hosts, which may indirectly influence thermal tolerance. Only manipulative experiments will allow us to fully understand the role of gregarines in hosts. In any case, our data unambiguously show that gregarines do not harm host thermal tolerance and could be considered mutualistic endosymbionts.

Overall, we suggest that gregarine endosymbionts do not reduce host thermal tolerance and may be associated with increased thermal tolerance. Although our findings are correlational, they suggest that gregarine infections could provide damselflies and other insects with some capacity to withstand rising temperature and frequent heat extremes. Understanding these host–endosymbiont interactions will be crucial to effectively predict the species’ vulnerability.

## Data Availability

The data that support the findings of this study and the codes used to analyse it are deposited in Figshare (https://figshare.com/s/e46a7b206c6c95e834bb).
